# Recent understanding of the pathophysiology of functional dyspepsia: role of the duodenum as the pathogenic center

**DOI:** 10.1007/s00535-019-01550-4

**Published:** 2019-02-14

**Authors:** Hiroto Miwa, Tadayuki Oshima, Toshihiko Tomita, Hirokazu Fukui, Takashi Kondo, Takahisa Yamasaki, Jiro Watari

**Affiliations:** Division of Gastroenterology, Department of Gastroenterology, Hyogo College of Medicine, Mukogawa-cho 1-1, Nishinomiya, Hyogo 663-8501 Japan

**Keywords:** Dyspepsia, Duodenum, Permeability, Microinflammation, Pathophysiology

## Abstract

Over almost 30 years since functional dyspepsia (FD) was defined, researchers have endeavored to elucidate the pathophysiology of functional gastrointestinal disorders. Now a consensus is emerging that the gastric symptoms of FD are caused mainly by gastric motility abnormalities and gastric hypersensitivity. The involvement of other causative factors including acid, *Helicobacter pylori*, psychological factors, and diet has been debated, but how they are involved in the manifestation of dyspeptic symptoms remains unclear. We believe that most of those factors cause FD symptoms by inducing gastric motility abnormalities and gastric hypersensitivity via the duodenum. Here, we discuss 2 possible reasons why patients with FD experience chronic upper abdominal symptoms: (1) the possibility that the contents of the duodenum of patients with FD differ from those of healthy persons and the different contents stimulate the duodenum, and (2) the possibility that the duodenum of patients with FD is more sensitive to noxious stimuli because of low-grade inflammation and increased mucosal permeability.

## Introduction

Functional dyspepsia (FD) is an extremely common disorder in which upper abdominal symptoms such as stomachache and heavy feeling in the stomach occur despite the absence of organic disease [1]. FD decreases the quality of life of patients suffering from it and the productivity of society. Until recently, most patients with FD were treated with antiulcer drugs and mucoprotective agents under the disease name chronic gastritis. However, with society’s increasing concern about quality of life, the trend of diagnosing and treating dyspepsia on the basis of its symptoms themselves has strengthened, and the disease name functional dyspepsia has come into use. Despite the adoption of this new name, the effectiveness of FD treatments has remained limited [2–4] because the pathophysiology of FD still is not well understood. Further elucidation of that pathophysiology may lead to new treatments. Over almost 30 years since non-ulcer dyspepsia was defined by the American Gastroenterological Association in 1989, the mechanism of dyspepsia symptom manifestation has been gradually elucidated, and in a recent advance, the idea that the duodenum may be the pathogenic center of FD was born.

## Understanding of the pathophysiology of functional dyspepsia to date

The vague nature of FD is probably one factor preventing the full elucidation of its pathophysiology; precisely defining FD is very difficult. Every human experiences dyspeptic symptoms, but such symptoms alone do not constitute a clinical disorder. The essence of FD is that the dyspeptic symptoms are experienced chronically, but chronically does not mean continuously. Rather, it means that FD patients experience dyspeptic symptoms more frequently than do healthy persons. For example, according to the Rome criteria, chronic is defined as fullness or early satiety 2 or more times per week, or epigastric pain one or more times per week, for at least 3 months [1]. Because the manifestation of FD symptoms is triggered by fatigue and stress, it is thought that abnormalities in the individual’s response to stress are part of the essence of FD [5]. But what determines the individual’s response to stress? Various causative factors have been proposed for FD, and many of them have been shown to be related to its pathophysiology either directly or indirectly, so FD is considered to be a multifactorial disorder.

Visceral hypersensitivity and abnormal gastric motility are thought to be the physiological abnormalities that directly cause FD symptoms, while factors including increased gastric acid secretion, *Helicobacter pylori* infection, psychophysiological abnormalities, diet, lifestyle, and stomach shape are thought to modify the expression of FD symptoms by inducing those physiological abnormalities. Patients with FD are thought to have abnormal individual responsiveness to stress, and that responsiveness is thought to be affected by factors including the environment from early childhood to adolescence, genetic abnormalities, and residual inflammation after gastrointestinal (GI) infection. It has also been reported recently that dysbiosis may be related to abnormal individual responsiveness. Interacting in a complicated manner, the above-mentioned factors induce dyspeptic symptoms. However, why the gastric physiological abnormalities are induced, how FD symptoms are modified, and why GI infection and other factors are related to sensitivity to stress remain unknown. In other words, the essence of the pathophysiology of FD remains unknown.

**Fig. 1:**
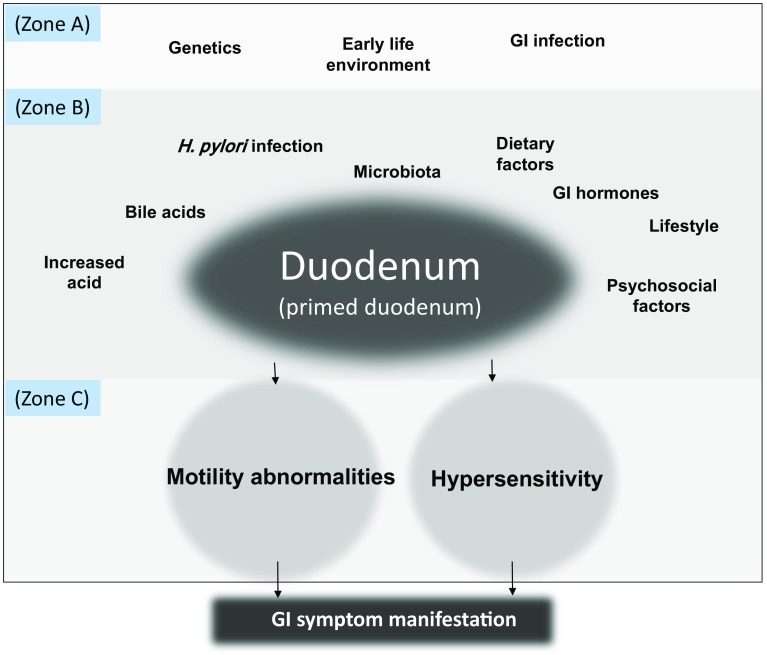
Although excessive response to stress is thought to underlie the pathophysiology of functional gastrointestinal (GI) disorders, there are factors that cause the excessive response. As we previously proposed, we think that the factors that contribute to manifestation of symptoms by patients with functional dyspepsia (FD) can be broadly divided into 3 categories. Zone A includes genetics, GI infections, and the environment early in life, especially stressful events; Zone C consists of the physiological abnormalities that directly cause FD symptoms, namely gastric motility abnormalities and gastric hypersensitivity; and Zone B includes numerous other factors—such as *Helicobacter pylori* infection, gastric acid, and diet—that modify the Zone C factors by acting on the duodenum. In this model, the duodenum can be thought of as the pathogenic center of FD

For some time, we have suspected that the above-mentioned factors do not independently cause FD symptoms, but rather contribute to symptom manifestation through a mechanism in which they interact [5]. We now believe that the duodenum is at the center of those interactions and is the key organ in the pathophysiology of FD, with various factors causing FD symptoms through their effects on it, and we have proposed dividing those factors into 3 categories: factors that govern abnormal responses to stress (Zone A), physiological abnormalities that directly induce symptoms (Zone C), and factors that modify those physiological abnormalities (Zone B) (Fig. 1). In summary, we believe that in patients with FD, stimuli to the duodenum induces gastric malfunction, which then causes dyspeptic symptoms.

## The duodenum and gastric physiological function

Although gastric motility abnormalities and gastric hypersensitivity are the factors thought to be directly related to manifestation of dyspeptic symptoms, both of those factors are known to be induced by stimulation of the duodenum. Indeed, there have been many reports that infusion of acid or lipid into the duodenum induces dyspeptic symptoms [6–10]. For example, using a barostat to investigate changes in gastric sensorimotor function caused by duodenal acidification in 10 healthy volunteers, Lee et al. found that infusion of 0.1 N hydrochloric acid into the duodenum induced proximal gastric relaxation, increased sensitivity to gastric distension, and inhibited gastric accommodation to a meal [11]. This experiment clearly showed that stimulation of the duodenum by acid induced gastric motility abnormalities and hypersensitivity and those physiological abnormalities caused dyspeptic symptoms. This suggests that the duodenum may be the center that controls the physiological functions of the stomach. However, while dyspepsia normally does not occur in healthy persons, it occurs chronically or as a result of minor stimulation in patients with FD. Perhaps the duodenums of patients with FD are different from those of healthy persons.

If the duodenum is the reason why dyspeptic symptoms occur differently in patients with FD than in healthy persons, we can consider 2 possibilities: (1) the possibility that patients with FD have an abnormal duodenal environment that somehow facilitates the entry of stimulants into the duodenum and thereby causes differences from healthy persons in the substances present in the duodenum, such as acid, bile acids, enteric bacteria, and lipids, and (2) the possibility that the duodenums of patients with FD are more sensitive to stimulation, and consequently, such patients react excessively to stimuli that do not provoke a reaction in healthy persons. The second possibility, in other words, is that the duodenal mucosa of patients with FD has been primed.

## Is the duodenal environment different in patients with FD?

First, we will consider the possibility that patients with FD may have an abnormal duodenal environment. Infusion of acid into the duodenum induces dyspeptic symptoms, but do patients with FD actually have higher levels of acid in their duodenum? Generally, the acid-neutralizing ability of the duodenum is extremely high, so duodenal acidification is unlikely to occur. Comparing gastric acid output in patients with FD with that in healthy persons, Collen and Loebenberg found that the patients did not have especially high levels of acid secretion [12]. Lee et al., however, found that postprandial duodenal pH was significantly lower in patients with relatively severe FD than in healthy controls [13]. Monitoring duodenal pH by radiotelemetry for 48 h in patients with FD and healthy controls, Bratten and Jones found that the patients had increased duodenal acid exposure after meals and during the daytime, and that reduced duodenal pH was correlated with early satiety [14]. Although it is difficult to explain why duodenal pH in patients with FD is decreased even though acid secretion is not especially increased, Samsom et al. found that when acid was infused into the duodenum of patients with FD and healthy controls, duodenal motility and acid clearance were decreased in the patients as compared with the controls [15]. Given the above findings, we cannot rule out the possibility that duodenal acidification causes dyspeptic symptoms.

**Fig. 2:**
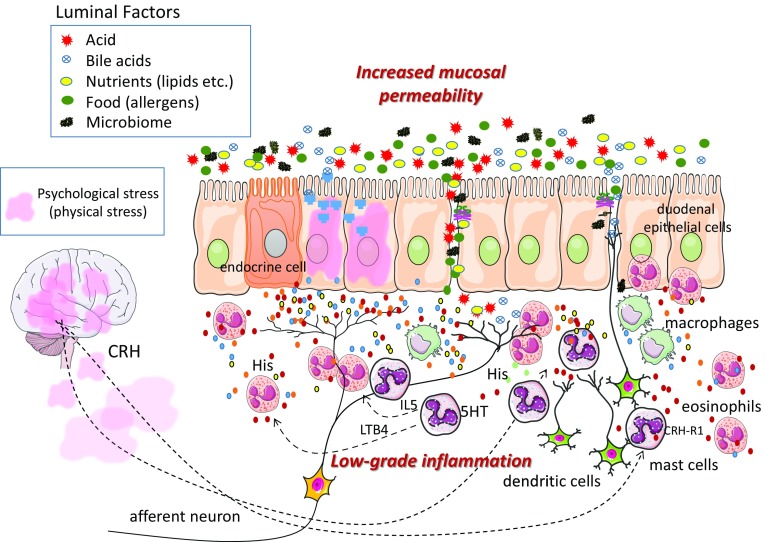
Duodenal events that may be occurring in patients with FD are shown schematically. Dyspepsia is caused by gastric motility abnormalities and gastric hypersensitivity that result from transmission of noxious stimuli from the duodenum by afferent nerves. The duodenums of patients with FD are thought to be susceptible to such stimulation. Associated with that susceptibility are two key pathogenic features—increased mucosal permeability and low-grade inflammation—which really are two sides of the same coin. The duodenal lumen has a wide variety of contents including acid, bile acids, lipids, food-derived substances, and enteric bacteria. Increased mucosal permeability would allow these contents to penetrate the mucosa, where they would be recognized by immune cells and then targeted by inflammatory cells. Such contents also might be able to stimulate submucosal afferent nerves and thereby cause physiological abnormalities in the stomach that result in dyspeptic symptoms. Duodenal contents also might directly stimulate duodenal mucosal cells, which as a result would then release inflammatory mediators that excite afferent nerves. Priming of the duodenum by low-grade inflammation caused by psychological stress or remaining after severe gastrointestinal infection (salmonellosis, dysentery, etc.) may be another important factor in FD pathogenesis. Because of the inflammation, sensory nerves would be sensitized by inflammatory mediators and cytokines released by eosinophils, mast cells, macrophages, and other inflammatory and immune cells, and mucosal permeability would be increased, making the duodenum more susceptible to effects from its contents. Thus, sensitization and priming of duodenal mucosa as a result of low-grade inflammation and increased mucosal permeability may be related to chronic FD symptoms

Acid, however, is not the only thing that is different in the duodenal environment of patients with FD. Recently, Beeckmans et al. reported that the bile acid components present in the duodenum may differ between patients with FD and healthy persons [16], and Tziatzios et al. hypothesized that overgrowth of bacteria in the duodenum may be involved in the pathogenesis of FD [17]. Thus, the duodenum of patients with FD may contain stimulants that are not present at the same levels in healthy persons. If that is true, the duodenal epithelium of patients with FD may be primed by constant exposure to such stimulants. Although there have not been many studies addressing the question of how the duodenal contents of patients with FD differ from those of healthy persons, the duodenal lumen contains a wide variety of potential stimulants including acid, bile acids, nutrients (lipids, amino acids, etc.), microorganisms, and potential allergens from food, and differences in the levels or composition of those maybe related to FD symptoms (Fig. 2). We are looking forward to further research on this question.

## Is the duodenum sensitized in patients with FD?

Although the duodenal contents of patients with FD do not differ much from those of healthy persons, it has been hypothesized that the duodenums of patients with FD are hypersensitive to stimulation. That is, even if duodenal stimulation is the same as that in healthy persons, patients with FD can overreact to that stimulation because of priming of their duodenal mucosa. This hypothesis is supported by a large amount of experimental evidence. For example, when lipids and other nutrients were infused into the duodenum, patients with FD had dyspeptic symptoms that were significantly more severe than those of healthy persons [18, 19]; those results show the excessive responsiveness of duodenal mucosa to nutrients. Patients with FD also had dyspeptic symptoms that were significantly more severe than those of healthy persons when hydrochloric acid was infused into the stomach [20]; this observation suggests that patients with FD may react excessively to acid that enters the duodenum from the stomach.

Although the mechanism of duodenal sensitization is not well understood, we think that increased permeability and microinflammation of duodenal mucosa are probably involved. Increased mucosal permeability would facilitate penetration of the mucosa by stimulants present in the duodenal lumen; then, after having directly penetrated the mucosa, such stimulants could stimulate afferent nerves and submucosal inflammatory cells. The presence of microinflammation indicates that the number of inflammatory cells in the mucosa or submucosa has increased, and such an increase would result in an increased amount of mediators released by those cells in response to stimulation. We also cannot forget that duodenal epithelial cells themselves could become primed. It is possible that repeated stimulation or inflammation causes the number of nociceptive receptors in duodenal epithelium to increase and their threshold value to decrease. Duodenal contents also may directly stimulate the duodenal epithelium and cause it to produce inflammatory mediators, but we have not seen reports of that in the literature so far.

## Increased permeability and microinflammation of duodenal mucosa

It seems possible that the duodenums of patients with FD are primed and that the causes are increased mucosal permeability and low-grade inflammation, but does that really occur? In a very interesting study, Vanheel et al. measured, using chamber, mucosal permeability in duodenal biopsy specimens from 15 patients with FD fulfilling the Rome III criteria and 15 age- and gender-matched healthy volunteers, and found that mucosal permeability was significantly greater in the patients with FD [21]. In addition, Ishigami et al. measured mucosal admittance, which is inversely correlated with mucosal impedance, in patients with FD and healthy volunteers, and found that duodenal mucosal permeability was significantly increased in the patients [22].

Generally, microinflammation and increased mucosal permeability are so closely related they can be thought of as two sides of the same coin. In other words, increased permeability of duodenal mucosa suggests that inflammation is present in the duodenum. Indeed, increased mucosal permeability, changes in tight junction proteins, and other pathological findings caused by slight inflammation have been reported in GI disorders including inactive ulcerative colitis, Crohn’s disease, and other inflammatory bowel diseases [23–25], *H.* *pylori* gastritis [26], and non-steroidal anti-inflammatory drug-induced damage [27]. There also have been many reports concerning microinflammation in the duodenum of patients with FD. In studies done in Sweden [28, 29] and the United Kingdom [30], eosinophilic infiltration of duodenal mucosa was found to be increased significantly in patients with FD, as compared with controls [28–30]. In another study, not only eosinophils, but also mast cells, were found to be increased in the duodenums of patients with FD [21]. Table 1 summarizes duodenal inflammation-related findings in patients with FD. As one would expect, other inflammatory cells such as macrophages and lymphocytes are also observed. We have found that eosinophils and mast cells are significantly increased in the duodenums of patients with FD, in particular, the second portion of the duodenum, and that those increases are correlated (data not shown, [38]). However, that cellular infiltration is not so extensive as to be obvious on microscopic observation; rather, when inflammatory cells in mucosal tissue are carefully counted in patients with FD and healthy persons, the patients have significantly higher counts. Again, inflammation of the duodenal mucosa in patients with FD is very slight.

**Table 1:** Duodenal low-grade inflammation in FD

Study (year)	Control (*n*)	FD (*n*)	Eosinophil		Mast cell	
			Infiltration	Degranulation	Infiltration	Degranulation
Talley et al. (2007) [28]	48	51	D1 ↑, D2 ↑	D1 ↑, D2 ↑	–	–
Walker et al. (2009) [29]	48	51	D1 ↑, D2 ↑	–	D1 →, D2 →	–
Walker et al. (2010) [30]	89	PDS 19	D ↑	–	–	–
Futagami et al. (2010) [31]	20	PI-FD 35	D ↑	–	–	–
Binesh et al. (2013) [32]	27	25	D →	–	D →	–
Walker et al. (2014) [33]	22	33	P-S: D1 ↑, D2 ↑	–	–	–
			PD-S: D1 →, D2 ↑			
Vanheel et al. (2014) [21]	15	15	D2 ↑	–	D2 ↑	–
Cirillo et al. (2015) [34]	20	18	D ↑	–	D ↑	–
Wang et al. (2015) [35]	39	141	D1 →, D2 ↑	D1 →, D2 ↑	D1 ↑, D2 ↑	D1↑, D2 ↑
Tanaka et al. (2016) [36]	5	9	D2 ↑	–	D2 →	–
Du et al. (2016) [37]	24	96	D1 →, D2 ↑	D1 ↑, D2 →	D1 →, D2 →	D1 →, D2 →
Taki et al. (2017) [38]	31	35	D2 ↑	–	D2 ↑	–

*FD* functional dyspepsia, *D* duodenum, *D1* bulb, *D2* s portion, *PI* post-infectious, *PDS* postprandial distress syndrome, *EPS* epigastric pain syndrome, *P*-*S* pain symptom, *PD*-*S* postprandial symptom

↑, increased; →, unchanged; –, not examined

Focusing on increased mucosal permeability and low-grade inflammation, Fig. 2 schematically illustrates the events that are thought to be occurring in the duodenums of patients with FD.

## Why do increased permeability and microinflammation occur in duodenal mucosa?

Why increased mucosal permeability and microinflammation are seen in the duodenal mucosa of patients with FD is an interesting question that is deeply related to the pathology of FD. Although there is no theory that clearly explains these phenomena, there is evidence for some possible explanations. One possible explanation is that inflammation may remain after a GI infection in some persons. FD in such persons is called post-infectious FD. In a cohort study, Mearin et al. surveyed participants about their symptoms before and after an outbreak of *Salmonella* gastroenteritis that occurred in Spain in 2002, and found that the risk for development of FD was about 5 times greater in the persons who had experienced acute gastroenteritis caused by *Salmonella enteritidis* during the outbreak than in controls [39]. Patients with dyspepsia increase not only after *Salmonella* gastroenteritis, but also after acute infection with *Escherichia coli* O157 or *Campylobacter jejuni* [40]. This explanation for the presence of microinflammation in patients with FD is also supported by the finding that the duodenal mucosa of patients with post-infectious FD contained higher levels of inflammatory cells and immune cells [31, 41]. The above evidence suggests that at least in some cases, microinflammation of duodenal mucosa may be residual inflammation remaining from acute GI inflammation.

There is also evidence that mast cell-mediated effects of psychological stress may increase mucosal permeability. In an experimental study, Vanuytsel et al. subjected healthy volunteers to different forms of psychological stress and measured the effect on intestinal mucosal permeability using the lactulose–mannitol ratio as an index [42]. One of the forms of stress was a public speech in which the participants presented their own research to a large group of people. Interestingly, this form of psychological stress caused mucosal permeability to increase. At the same time, the study also showed that administration of corticotropin-releasing hormone (CRH) increased mucosal permeability, and that pre-administration of the mast cell stabilizer disodium cromoglycate suppressed both the speech- and CRH-induced increases in mucosal permeability. On the basis of those findings, the authors suggested that mast cells may be involved in increased mucosal permeability induced by stress.

It is also possible that dysbiosis may increase mucosal permeability. Xu et al. reported that water avoidance stress caused rectal hyperalgesia and increased mucosal permeability in rats, and that pre-administration of the poorly absorbed antibiotic rifaximin prevented the increase in mucosal permeability and altered the composition of bacterial communities in the ileum [43].These results suggest that changes in enteric bacteria may increase intestinal mucosal permeability.

Thus, it has been reported that residual inflammation after acute GI infection and acute psychological or physical stress may increase intestinal mucosal permeability, but further research will be necessary to fully determine the stimuli that increase intestinal permeability.

## The duodenum as the pathogenic center of functional dyspepsia

Dyspepsia occurs because noxious stimuli transmitted from the duodenal mucosa by afferent nerves cause gastric motility abnormalities and gastric hypersensitivity. Compared to healthy persons, patients with FD may be more prone to dyspeptic symptoms because their duodenum has become more sensitive to stimulation; that is, it has become primed. Microinflammation and increased permeability of duodenal mucosa have been inferred to cause that priming, and studies have shown that microinflammation can remain after GI infection and psychological stress can increase mucosal permeability. On the other hand, one cannot rule out the possibility that the duodenums of patients with FD are more excitable than those of healthy persons because the duodenal contents of patients with FD have greater stimulatory potential than those of healthy persons; however, there have not been many studies addressing the question of such differences in duodenal contents. Questions for future research include “What causes duodenal inflammation?” and “How are *H. pylori* and the other risk factors that have been proposed as causes of FD related to duodenal inflammation?” Early life trauma is a well-known risk factor for FD, but whether psychological trauma is involved in microinflammation or increased permeability of GI mucosa remains an interesting question, as does how the intestinal microbiota is related to duodenal inflammation.

## Abbreviations

FD: Functional dyspepsia
GI: Gastrointestinal
CRH: Corticotropin-releasing hormone
